# Prognostic factors for persistent symptoms in adults with mild traumatic brain injury: protocol for an overview of systematic reviews

**DOI:** 10.1186/s13643-021-01810-6

**Published:** 2021-09-23

**Authors:** Julien Déry, Élaine De Guise, Ève-Line Bussières, Marie-Eve Lamontagne

**Affiliations:** 1 Department of Rehabilitation, Université Laval, Pavillon Ferdinand-Vandry, Local 2475, 1050, Avenue de la Médecine, Québec, G1V 0A6 Canada; 2 Centre interdisciplinaire de recherche en réadaptation et intégration sociale (CIRRIS), Centre intégré universitaire de santé et de services sociaux de la Capitale-Nationale, 525, boul. Wilfrid-Hamel, Québec, G1M 2S8 Canada; 3 Department of Psychology, Université de Montréal, Montréal, Canada; 4 Research Institute of the McGill University Health Centre (RI-MUHC), Montréal, Canada; 5 Centre de recherche interdisciplinaire en réadaptation du Montréal métropolitain (CRIR), Montréal, Canada; 6 Department of Psychology, Université du Québec à Trois-Rivières, 3007 Michel-Sarrazin, 3600 rue Sainte-Marguerite, Trois-Rivières, Québec, G9A 5H7 Canada

**Keywords:** Overview, Systematic review, Prognosis, Mild traumatic brain injury, Persistent symptoms

## Abstract

**Background:**

Mild traumatic brain injury (mTBI) is an increasing public health problem that can lead to persistent symptoms that have several functional consequences. Understanding the prognosis of a condition is an important component of clinical decision-making and can help guide the prevention of long-term disabilities of patients with mTBI. Several studies and systematic reviews have been conducted in order to understand prognosis of chronic symptoms following mTBI. We aim to synthesize evidence from systematic reviews on factors that affect the risk of persistent symptoms in mTBI-affected adults.

**Methods:**

We will conduct an overview of systematic reviews following steps described in the Cochrane Handbook. We will search in Cochrane, Medline, CINAHL, Embase, PsycINFO, and Epistemonikos for systematic reviews about the prognosis of persistent symptoms following mTBI in the adult population. Two reviewers will independently screen all references and then select eligible reviews based on eligibility criteria. A data extraction grid will be used to extract relevant information. The risk of bias in the included reviews will be assessed using the ROBIS tool. Data will be synthesized into a comprehensive conceptual model in order to have a better understanding of the predictive factors of post-concussion symptoms following mTBI.

**Discussion:**

Results will help multiple stakeholders, such as clinicians and rehabilitation program managers, to understand the prognosis of long-term consequences following mTBI. It could guide stakeholders to recognize their patients’ prognostic factors and to invest their time and resources in patients who need it the most.

**Systematic review registration:**

PROSPERO CRD42020176676.

**Supplementary Information:**

The online version contains supplementary material available at 10.1186/s13643-021-01810-6.

## Background

Cases of mild traumatic brain injury (mTBI) have increased in recent years [1, 2], and their long-lasting effects have received increasing attention [3]. Most of the symptoms disappear in a few days or weeks [4], but 5% to 20% of sufferers encounter persistent physical, cognitive, and behavioral disabilities [5]. The most prevalent chronic symptoms are headaches, difficulty concentrating, fatigue, and dizziness [6]. These chronic symptoms can have important impacts on day-to-day activities and can lead to functional consequences such as delays in return to work [7, 8]. To prevent persistent symptoms of mTBI, it is important to identify and understand factors that can interfere with the symptomatology, such as depression, chronic pain, and situational stress [5]. To recognize symptoms and to diagnose mTBI is a good start, but identifying what contributes to persistent symptoms in the target population would help healthcare providers to target the right patients, and even prioritize them based on these prognostic factors, before symptoms become chronic.

Understanding a condition’s prognosis is a central component of clinical decision-making [9]. Many factors can be related to a positive or poor prognosis in a given condition. Identifying prognostic factors that can contribute to poor prognosis in mTBI is important in order to better understand this condition and to target patients with the greatest needs [9]. Patients with a poor prognosis should arguably be considered for more in-depth evaluations and targeted interventions in the early stages of the condition in order to prevent transitioning to chronicity. In fact, persistent disability from mTBI could be reduced by identifying and addressing earlier risk factors, such as comorbidities (e.g., depression, and anxiety) [10, 11]. Long-lasting symptoms after mTBI are both complex and nonspecific, which highlights the need to identify subgroups of the mTBI population that might benefit from specific and early interventions [11].

Over the past years, several studies and reviews have focused on possible factors contributing to persistent disabling problems after suffering mTBI. The World Health Organization (WHO) Collaborating Centre Task Force has produced an extensive number of systematic reviews related to mTBI prognoses [2–4, 11–18]. These reviews mainly synthesized studies employing longitudinal designs to identify time to recovery and prognostic factors affecting symptom persistence [14]. Numerous and diverse factors were identified as predictors of prolonged symptoms, such as financial compensation, being married, being off work due to the injury, post injury symptoms of nausea or memory problems, and other injuries (percentage of the body in pain after the collision), pre-existing physical limitations, prior brain illness or neurological problems, prior head injuries, psychiatric problems, life stressors, being a student, sustaining mTBI in a motor vehicle collision, and age over 40 years [14].

**Fig. 1:**
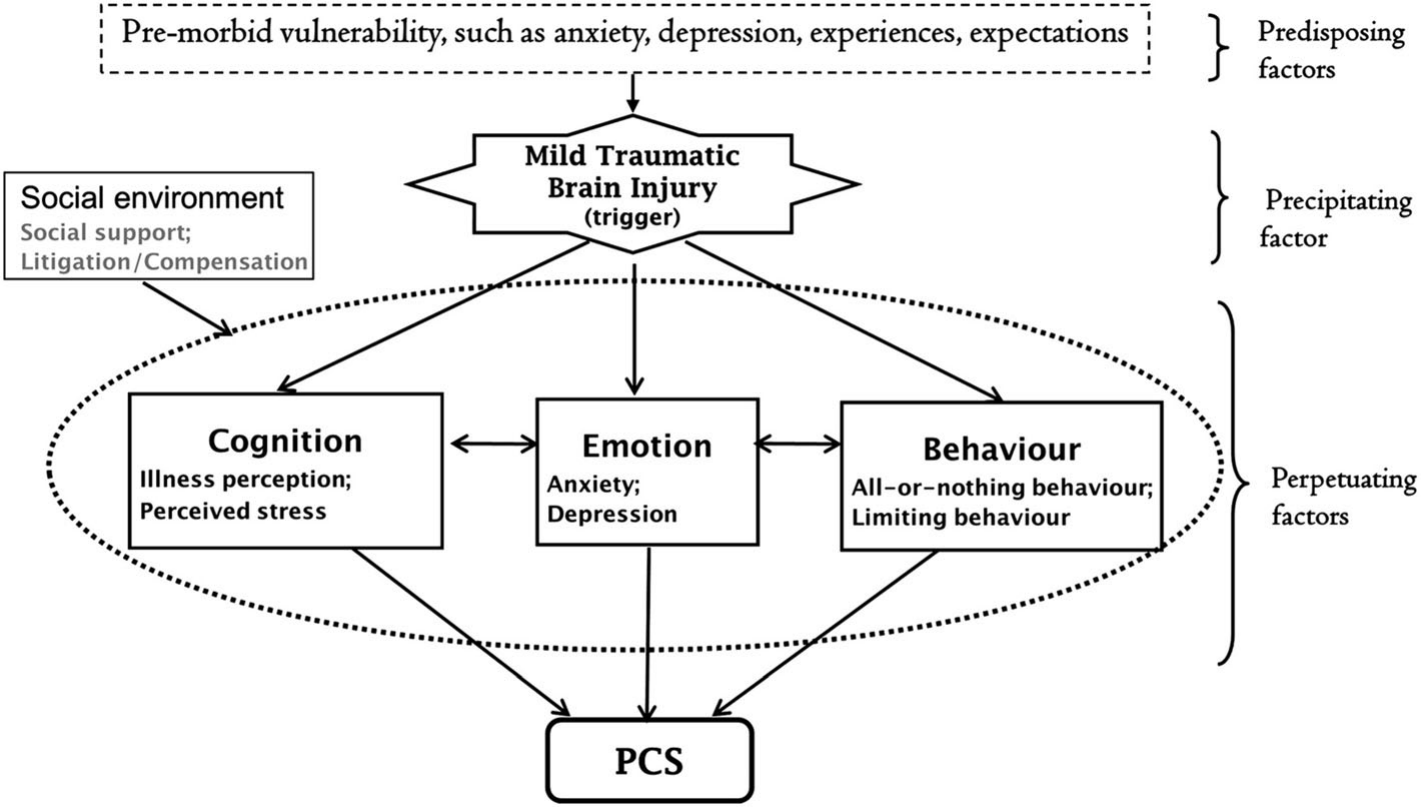
The proposed model of postconcussional syndrome (PCS) by Hou et al. [26]

Systematic reviews on the subject of prognosis after mTBI have targeted different populations, such as workers [19], athletes [20], military [12], and even a mix of all populations [21]. While some reviews focused their research question on particular prognostic factors including sex [13], age [22, 23], and biomarkers [24, 25], others were interested in specific outcomes related to mTBI, such as cognitive and psychiatric outcomes [4, 17]. Healthcare decision-makers including healthcare providers, policy makers, researchers, funding agencies, informed patients, and caregivers may have difficulties in fully understanding information from these multiple systematic reviews and what factors contribute specifically to persistent disabilities. Thus, a comprehensive synthesis could inform stakeholders regarding prognostic factors of persistent symptoms after mTBI. The model proposed by Hou et al. [26] highlighted that potential risk factors contributing to persistent symptoms (postconcussion symptoms) are an interaction between biological, psychological, and social factors. As shown in Fig. 1, the risk factors are characterized as predisposing factors (e.g., having a premorbid personality trait of anxiety proneness and/or a history of somatic complaints); precipitating factors (e.g., related to the brain injury); and perpetuating factors (e.g., cognitive, behavioral, and emotional reactions to the injury).

We aim to combine all prognostic factors into a conceptual model in order to have a usable and comprehensive document to clearly address the predictors of chronic symptoms that contribute to long-term disabilities in the adult population having experienced mTBI. Considering the diversity of published systematic reviews, we argue that there is sufficient up-to-date evidence to conduct a synthesis of systematic reviews and to integrate results into a comprehensive model, such as the one presented by Hou et al. [26]. We wish to synthetize evidence in a way that will make it understandable to the end-user and allow presentation of the individual systematic reviews in a single product [27].

An overview of systematic reviews is a rigorous approach to mapping evidence of a large body of literature in a given area [28, 29]. Nomenclature and methods used for this type of review vary in the literature: “umbrella reviews,” “meta-reviews,” “overviews of systematic reviews,” “reviews of reviews,” and “a systematic review of systematic reviews,” among others [30]. The approach requires similar search strategies and quality/risk of bias scoring as a systematic review of primary literature, but the unit of searching, inclusion, and data extraction is the systematic review (and not the primary study) [27, 31].

## Objectives

The objective of this research study is to synthesize evidence from two or more systematic reviews on factors that affect the risk of persistent symptoms in adults with mTBI. We aim more specifically to gather information about (1) predisposing factors; (2) precipitating factors; and (3) the contribution of cognitive, emotional, behavioral, and social perpetuating factors in the development of persistent postconcussion symptoms. Objectives may be modified or refined as our knowledge of the underlying systematic reviews evolves, but all modifications will be documented and accompanied by a rationale in the final review article.

## Methods

We will conduct an overview of systematic reviews according to the principles of the Cochrane Handbook’s chapter on methods for overviews of reviews [27] and other recent methodological papers [32–34]. Overviews of reviews (OvR) are designed to compile, synthesize, and integrate evidence from multiple systematic reviews into one accessible and usable document [32]. Despite their increasing popularity in healthcare research over the past years, there are currently no systematically developed reporting guidelines for OvR [30, 35]. However, OvR methods have well-established standards of conduct to ensure rigor, validity, and reliability of results [27]. Considering that our OvR aims to summarize the prognostic factors associated with changes in health outcomes, we consulted the PROGRESS (PROGnosis RESearch Strategy) framework to create this protocol [9, 36]. This protocol has been prepared in consultation with the PRISMA-P statement [37] (checklist provided in the supplemental documents to this protocol, in the additional file). This review is registered in the PROSPERO database (CRD42020176676). Any amendments to the protocol will be described in the final review article.

### Criteria for selecting reviews for inclusion

We will include systematic reviews with or without a meta-analysis. We will consider publications to be systematic reviews if they were explicitly described in the report as being based on a systematic search, and if the search strategy and the article selection process are precisely presented (e.g., a PRISMA flow diagram), with enough detail for them to be reproducible. The following were considered as defining features of a systematic review in our overview [38]:

- Clearly articulated objectives and questions to be addressed
- Inclusion and exclusion criteria, stipulated a priori (in the protocol), that determine the eligibility of studies
- A comprehensive search to identify all relevant studies, both published and unpublished
- Appraisal of the quality of included studies, assessment of the validity of their results, and reporting of any exclusions based on quality
- Analysis of data extracted from the included research
- Presentation and synthesis of the findings extracted
- Transparent reporting of the methodology and methods used to conduct the review

Systematic reviews that include randomized controlled trials or variable study designs such as observational studies will be included. We will consider reviews targeting only adult populations (18 years of age and over) with mTBI or concussion. Reviews that targeted multiple populations (e.g., children and adults) will be included, but only primary studies that were conducted in adults will be analyzed in the overview. To have a common definition of the condition, we will adhere to the WHO Collaborating Centre Task Force on Mild Traumatic Brain Injury definition [39]:

MTBI is an acute brain injury resulting from mechanical energy to the head from external physical forces. Operational criteria for clinical identification include: (1) 1 or more of the following: confusion or disorientation, LOC for 30 minutes or less, posttraumatic amnesia for less than 24 hours, and/or other transient neurologic abnormalities such as focal signs, seizure, and intracranial lesion not requiring surgery; (2) GCS score of 13–15 after 30 minutes post-injury or later upon presentation for health care. (3) These manifestations of MTBI must not be due to drugs, alcohol, medications, caused by other injuries or treatment for other injuries (e.g., systemic injuries, facial injuries, or intubation), caused by other problems (e.g., psychological trauma, language barrier, or coexisting medical conditions), or caused by penetrating craniocerebral injury.

We will not restrict our inclusion process based on a specific setting or context, e.g., patients from primary care, hospitals, and rehabilitation care. No specific eligible time period will be considered in the inclusion criteria. We will include articles that reviewed the course of persistent postconcussion symptoms, i.e., “presence of any symptom that cannot be attributed to a preexisting condition and that appeared within hours of an mTBI, that is still present every day 3 months after the trauma, and that has an impact on at least one sphere of a person’s life” [40]. Narrative, non-systematic reviews, editorials/commentaries, or gray literature, such as thesis work, will not be eligible for this OvR. We will also exclude reviews about moderate, severe, or non-traumatic brain injuries, and reviews targeting only child populations (under 18 years old).

### Search methods for identification of reviews

A professional librarian and members of the research team helped to develop the search strategy based on the objective of the review. We used the PECO-S model to develop our search strategy, as proposed by Morgan et al. [41]: (1) Adults with mTBI/concussion (population); (2) presence of any risk or prognostic factors (exposure); (3) absence of exposure (comparison); (4) any persistent symptoms (outcomes); and (5) systematic reviews (study type). We chose to add a “study type” concept considering that we will focus our search on systematic reviews only. In addition, we decided to include only concepts 1 and 5 in our search to improve its sensitivity and to avoid exclusion of pertinent reviews. We will search in five relevant databases (Cochrane (Wiley), Medline (Ovid), CINAHL (EBSCO), Embase (Elsevier), PsycINFO (Ovid)) and Epistemonikos for systematic reviews published in peer-reviewed journals without date restrictions. We validated our strategy by consulting search filters such as those reported by the InterTASC Information Specialists’ Sub-Group [42]. An example of a search strategy in Medline (Ovid) is displayed in Table 1.

**Table 1:** Example of search strategy in Medline (Ovid) database

1) Concept: **mTBI concussion**		
#1	[ti.]	(concuss* OR commotio* cerebr* OR cerebral* commotio* OR mtbi) or ((mild OR minor OR minimal) adj3 (traumatic brain OR tbi))
#2	[ab.]	(concuss* OR commotio* cerebr* OR cerebral* commotio* OR mtbi) OR ((mild OR minor OR minimal) adj3 (traumatic brain OR tbi))
#3	[Mesh]	Brain injuries, traumatic/
#4	[Mesh]	Brain concussion/
#5	#1 OR #2 OR #3 OR #4	
2) Concept: **Systematic reviews**		
#6	[pt.]	Systematic review
#7	**#5 AND #6**	

### Selecting systematic reviews for inclusion

All references will be imported from the databases into reference management software (Endnote). Duplicates will be removed using the software command “Find duplicates” based on the titles of the references. Once duplicates have been removed, the screening and selection process will be performed using the Covidence online software [43]. Two reviewers (JD, MEL) will independently screen all references identified from the literature search. References judged potentially eligible after screening of titles and abstracts will be reviewed in full-text form. Any disagreements will be discussed by the two reviewers, who will consult a third member of the team if consensus is not reached. If only a subset of primary studies contained within certain systematic reviews meets the eligibility criteria, we will include the reviews in the OvR, but we will exclude irrelevant primary studies from the analysis. After identifying all systematic reviews that meet the inclusion criteria, we will not conduct supplementary searches for primary studies [27].

### Managing overlapping systematic reviews

We acknowledge the risk that the systematic reviews included may address similar research questions and include many of the same primary studies; this is a factor that we will take into consideration in the data analysis methods. If we find overlapping systematic reviews, we will avoid double-counting data by ensuring that each primary study’s findings are extracted separately (if available). As recommended by Pieper et al. [44], we will create a citation matrix to visually demonstrate the amount of overlap and we will calculate the “corrected covered area” (CCA), which is an indication of the degree of overlap in overviews.

### Data collection and analysis

Relevant information from all selected reviews will be grouped in an extraction grid (see supplementary files). We prepared a table with the characteristics of the included reviews and we will extract basic information about the systematic reviews (e.g., title; authors; year of publication; designs of the primary studies; number of primary studies included; and purpose of the reviews). We will also extract information about the systematic reviews’ search strategies (e.g., number, names and date ranges of databases searched; date of last search update) and population (e.g., participant characteristics). Key items related to studies of prognostic factors will be extracted from the reviews, such as participants, sample size, outcomes to be predicted, prognostic factors, the analysis conducted, and other relevant results [9]. Our extraction will focus on the outcomes related to persistent postconcussion symptoms, according to the definition proposed by Lagacé-Legendre et al. [40]. Extraction will be limited to information presented in the included systematic reviews, so we will not further examine the primary research studies [31]. Two independent reviewers (JD, MEL) will extract data and then will compare their respective grids and reach a consensus.

### Summarizing data

We will present information from the reviews about prognostic factors in narrative summaries. In order to group similar populations or outcome measures, we will present groups of similar systematic reviews and/or outcome measures together. This synthesis will be inspired by the model proposed by Hou et al. [26] which organized and classified the predictors in larger categories of prognostic factors, i.e., predisposing, precipitating, and perpetuating factors. We will present data in tabular form, as well as graphically if possible, in order to visually demonstrate the diversity of the data. We will produce a synthesized list of prognostic factors considering evidence quality, risk of bias, and the factors’ association with persistent symptoms. Narrative descriptions from each review will complement the data and provide a comprehensive understanding of the synthesis. Because systematic reviews of prognostic factors present their results in a form which does not permit re-analysis of primary data, we will not attempt to quantify prognostic effects in a risk prediction model [9, 36].

### Quality of included reviews

Risk of bias of the included reviews will be rated using the Risk of Bias in Systematic Reviews (ROBIS) tool [45], as recommended by Pollock et al. [27]. ROBIS is useful and reliable for systematic reviewers to identify areas where bias may be introduced in systematic review methods: study eligibility criteria; identification and selection of studies; data collection and study appraisal; and synthesis and findings [45]. We will summarize the number of systematic reviews that had low, high, or unclear concerns for each domain and the number of reviews at high or low risk of bias. We will display the summarized results of this assessment in a table and a figure (see additional files) as suggested by Whiting et al. [45]. Risk of bias ratings will be used in the data synthesis process to inform the conclusions of this review. No reviews will be excluded based on their respective risk of bias evaluations. We will extract the assessments (risk of bias and quality) that are presented in each included systematic review and then present them in narrative and/or tabular summaries.

## Discussion

There is increasing interest in the mTBI population and in the research concerning mTBI prevention, diagnosis, and prognosis [4, 14, 15, 46]. A large body of evidence has emerged concerning the course of this healthcare condition. Multiple systematic reviews have synthesized information about this population and many have studied prognostic factors associated with persistent symptoms.

The results of an OvR (systematic review of systematic reviews) of prognostic factors related to persistent symptoms after mTBI would provide a comprehensive state of the evidence in this area. We aim to present a systematic evidence synthesis concerning all factors that affect the risk of persistent symptoms and to describe the body of evidence that supports it. In order to have an organized and usable synthesis, our goal is to organize all prognostic factors into categories based on the types of factors found in the literature.

We expect that results of this OvR will help multiple stakeholders, such as clinicians and healthcare managers, to understand the prognosis of their patients and to focus their time and resources on patients who need it the most. The results of this overview could also inform decision-makers and policymakers of the importance of early identification of prognostic factors in order to prevent onset of persistent symptoms and their disabling consequences. Highlighting all the possibilities of prognostic factors that have been studied in adults with mTBI could benefit the researchers who wish to more deeply investigate the outcomes related to these factors.

We anticipate potential limitations of this overview, firstly concerning variety of the characteristics of the populations included in the reviews. Systematic reviews often regroup populations composed of veterans and military personnel and adults with sport-related brain injury. We expect to identify diverse prognostic factors and heterogeneity in the methods that could be difficult to synthesize into a single comprehensive model. Our methods will focus on descriptive analyses and a narrative synthesis which will take heterogeneity of the reviews into consideration. Overviews of reviews are often known for lacking methodological rigor because there are no pre-established reporting guidelines [47]. However, we have based our methods on the updated Cochrane Handbook [27] and several previous works [28–34] that can appropriately guide us through a rigorous process.

## Supplementary Information

**Additional file 1.** PRISMA-P 2015 Checklist.

**Additional file 2.** Characteristics of the systematic reviews included. Key results on prognostic factors.

**Additional file 3: Table.** Results of ROBIS assessment. **Figure.** Example of graphical presentation of ROBIS results.

## Data Availability

All data generated or analyzed during this study will be included in the published overview article.

## Abbreviations

CINAHL: Cumulative Index to Nursing and Allied Health Literature
mTBI: Mild traumatic brain injury
OvR: Overview of systematic reviews
PRISMA(-P): Preferred Reporting Items for Systematic Reviews and Meta-Analyses (Protocol)
PCS: Postconcussional syndrome
ROBIS: Risk of bias in systematic review
WHO: World Health Organization
