# Supplementary material for: Prognostic factors for persistent symptoms in adults with mild traumatic brain injury: protocol for an overview of systematic reviews
Source: Syst Rev. 2021 Sep 23;10:254. doi: 10.1186/s13643-021-01810-6 (PMC8461939; doi:10.1186/s13643-021-01810-6)
Supplement: Supplementary file 2 — Additional file 2. Characteristics of the systematic reviews included. Key results on prognostic factors. [file 13643_2021_1810_MOESM2_ESM.docx]

Characteristics of the systematic reviews included

| Reference of the review | # of primary articles | Designs of the studies included | Population | Goal of the review | ROBIS Assessment |
| --- | --- | --- | --- | --- | --- |
| Review 1 |  |  |  |  |  |
| Review 2 |  |  |  |  |  |
| … |  |  |  |  |  |

Key results on prognostic factors

| Review | Participants, sample size | Prognostic factor | Outcomes | Analysis conducted | Conclusions |
| --- | --- | --- | --- | --- | --- |
| Review 1 |  |  |  |  |  |
| Review 2 |  |  |  |  |  |
| … |  |  |  |  |  |
