# Supplementary material for: Prognostic factors for persistent symptoms in adults with mild traumatic brain injury: protocol for an overview of systematic reviews
Source: Syst Rev. 2021 Sep 23;10:254. doi: 10.1186/s13643-021-01810-6 (PMC8461939; doi:10.1186/s13643-021-01810-6)
Supplement: Supplementary file 3 — Additional file 3: Table. Results of ROBIS assessment. Figure. Example of graphical presentation of ROBIS results. [file 13643_2021_1810_MOESM3_ESM.docx]

**Table**. Results of ROBIS assessment

| Review | Concerns regarding specification of study eligibility criteria | Concerns regarding methods used to identify and/or select studies | Concerns regarding methods used to collect data and appraise studies | Concerns regarding methods used to synthesize results | Risk of bias in the review |
| --- | --- | --- | --- | --- | --- |
| Review 1 |  |  |  |  |  |
| Review 2 |  |  |  |  |  |
| … |  |  |  |  |  |

**Figure.** Example of graphical presentation of ROBIS results
